# The practical application of biomechanical theory for patient assessment

**DOI:** 10.1186/1757-1146-4-S1-I9

**Published:** 2011-05-20

**Authors:** Trevor D Prior

**Affiliations:** 1 Homerton University Hospital, London, E9 6SR, UK

## 

Podiatric biomechanical assessment has become a fundamental cornerstone of podiatric practice. However, whilst there are many papers indicating the positive benefits of orthotic intervention, the underlying assessment philosophies remain controversial. The work produced by Root, Orien and Weed still underpins much of the current assessment and management techniques, yet key aspects of their concepts are not supported by scientific scrutiny. For every paper that seems to support aspects of function, there are more which do not support common theory. As a result, several conceptual assessment techniques have been developed and expounded although the evidence base to support these theories remains weak at best. This leaves the clinician who wishes to practice evidenced based medicine in a dilemma when presented with a patient in discomfort. To date, there has been little evidence to guide such an approach and thus replace the disproved theory. This workshop will review common assessment techniques and factors which effect limb function. It will outline an approach which can be taken to integrate these techniques and provide a basis for care utilising the current evidence available. This will include a practical demonstration of assessment techniques.

